# Imatinib-induced pancreatitis

**DOI:** 10.4103/0253-7613.62407

**Published:** 2010-02

**Authors:** Mahesh R. Varma, Shibi Mathew, Devadas Krishnadas, K.R. Vinayakumar

**Affiliations:** Department of Medical Gastroenterology, Medical College, Thiruvananthapuram, India

**Keywords:** Acute pancreatitis, chronic myeloid leukemia, imatinib mesylate

## Abstract

Drug-induced pancreatitis is a rare but serious complication of many drugs, some of which have been well documented. Here we present a case of a middle-aged man with chronic myeloid leukemia who developed acute pancreatitis after being initiated on imatinib mesylate. The case history, the pharmacodynamics, uses, and adverse effects of imatinib mesylate are discussed in detail.

## Introduction

Acute pancreatitis (AP) is a well-known complication of many antineoplastic agents. Here we present a case of a 53-year-old man who developed AP while treated with imatinib mesylate. The development of AP after being initiated on imatinib is relatively uncommon. There are no more than 10 cases reported in the literature where imatinib-induced pancreatitis has been mentioned as a serious adverse event.

## Case Report

A 53-year-old man who had been diagnosed as having Chronic Myeloid Leukemia (CML) 10 months back and was in remission on imatinib mesylate, presented with a history of epigastric pain of 2 months duration which had worsened for the last 10 days. The pain was of steady, boring type with frequent waxing and waning, and related to food intake. There was no vomiting, gastrointestinal (GI) bleed, abdominal distension, ball rolling sensation, or increased borborygmi. He had sitophobia and was predominantly on semisolid food for the past 1 week. He used to consume around 10 units of hard liquor per week for 15 years (15–20 g/day), till 5 years back and is a chronic active smoker. There was no history suggestive of intermittent claudication or abdominal angina. Moreover, there was no history of jaundice or gallstone disease in the past. He was diagnosed as having CML with Ph(+) chromosome status after which chemotherapy with imatinib was started. He was on imatinib for 1 month when the pain started. He was also on metformin 500 mg BD and amlodipine 5 mg OD for the last 5 years for diabetes and hypertension.

His physical examination revealed anemia, with tenderness and fullness in the epigastrium. He was found to have massive splenomegaly. Other systemic examination was normal.

The patient had hemoglobin of 7.8 g/dL with indices suggestive of iron deficiency anemia. His total count was normal (7600/cu mm) with 67% neutrophils. His platelet count was 4.5 lakhs/cu mm and peripheral smear did not show evidence of blast crisis or accelerated phase of CML. Liver function tests revealed elevated alkaline phosphatase and low albumin with aminotransferases >150 u/mL. His prothrombin time and renal function were normal. The markers of inflammation were elevated with an ESR of 76 mm/h and a CRP value of 4 mg/dL (normal 0.2–0.6). The random blood sugar was 292 mg/dL Corrected calcium was normal. He had three out of seven of the Glasgow severity scores indicating severe AP. His serum lipase was elevated to a value of 3500 IU and later fell on serial examination. The arterial blood gas analysis revealed a normal pH with a pO2 of 83 mmHg.

**Figure 1:**
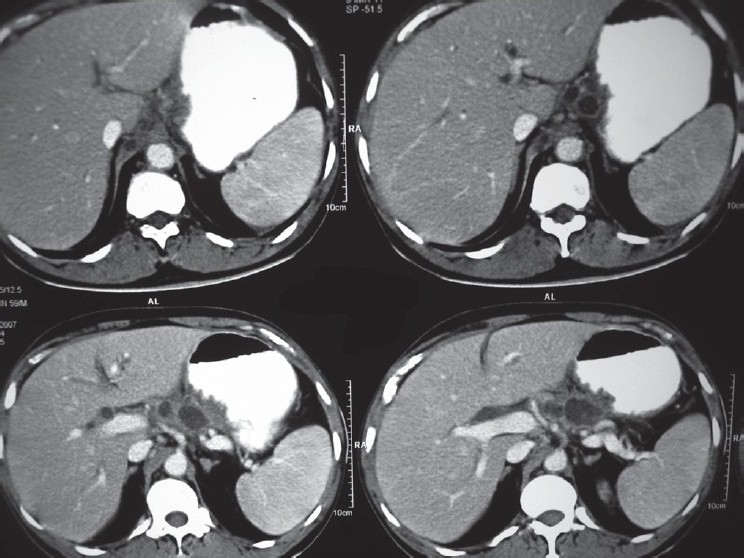
Contrast enhanced CT scan of the pancreas showing cystic lesions in the body and tail of pancreas. Splenomegaly is also noted.

**Figure 2:**
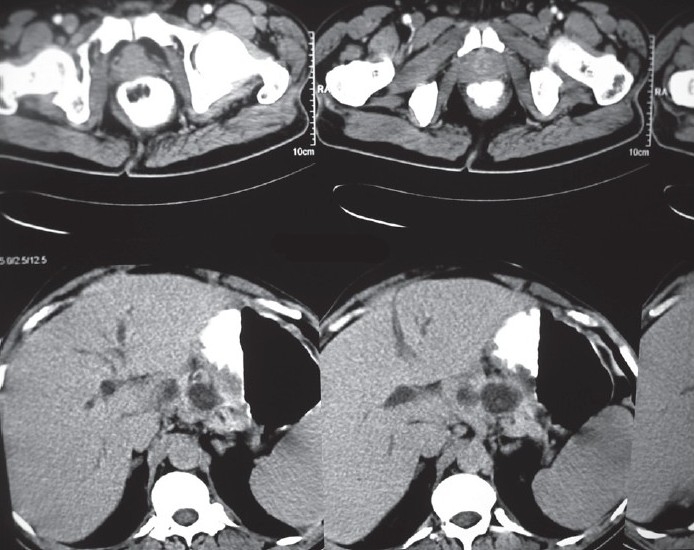
CT scan of the pancreas showing pseudocysts in the body and tail.

Imaging by abdominal multidetector CT revealed pseudo cysts of varying sizes in head and body regions of pancreas [Figure 1] with the largest cyst showing inhomogeneous opacities suggestive of infected/hemorrhagic cysts [Figure 2]. Common bile duct was dilated, and there was mild central intrahepatic biliary radical dilatation. Other abdominal organs were normal, and a diagnosis of AP with pseudocyst formation was made.

He had stopped consuming alcohol 3 years back; AP rarely occurs in alcoholic patients without underlying changes of chronic pancreatitis (i.e., a single “binge” is unlikely to result in AP).[1] The dose of at least 150 g/day (35 U/week) of intake exceeding 5 years are required before the development of “silent” chronic pancreatitis. In addition, this occurs only in 3–15% of those consuming alcohol.[2] The patient was on metformin 500 mg BD for the last 5 years. Pancreatitis occurred on starting the drug, and there was prompt relief of symptoms on stopping the drug. Further, there was no evidence of any co-factors that might have triggered the disease. Causality assessment of the adverse drug event by Naranjo's algorithm was conducted. A score of seven was obtained (causality—probable).[3] Considering that the drug imatinib was the last drug that had been started for the patient, it was considered the possible causal agent for producing pancreatitis. On admission, imatinib was stopped and the patient had marked relief of symptoms, the abdominal pain subsided and the lipase levels reduced.

**Figure 3:**
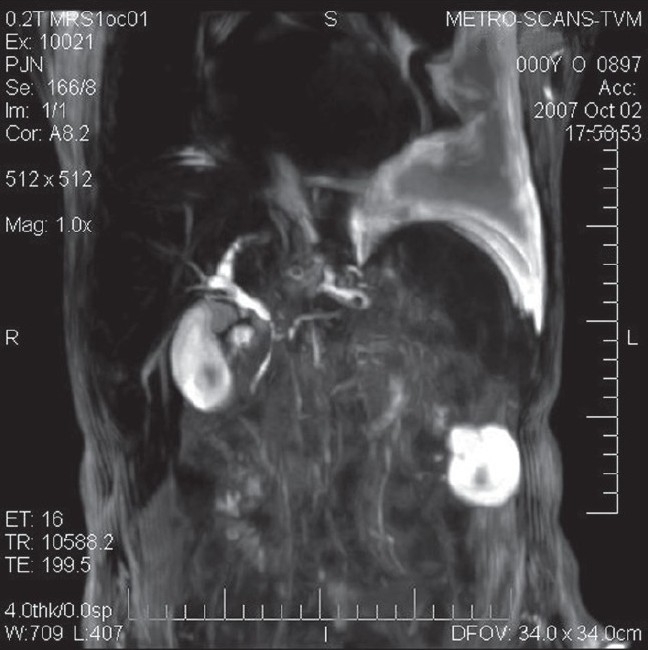
MRCP showing pancreaticopleural fistula with pleural effusion.

**Figure 4:**
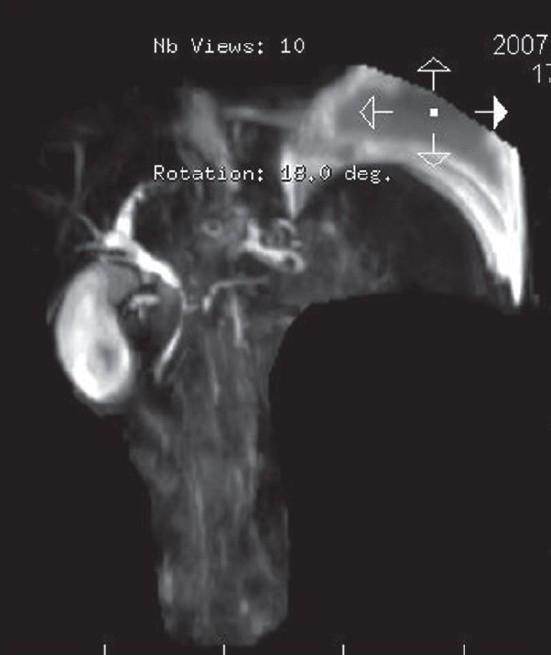
MRCP image showing a connection with pancreatic duct and the pleural space.

Two weeks later, the patient returned with a massive left-sided pleural effusion and high serum amylase (14400 IU/mL) and lipase (15000 IU/mL). He was subjected to repeated pleural fluid aspirations for respiratory compromise. The improvement was transient, and the patient developed rapid reaccumulation of pleural fluid. Magnetic Resonance Cholangio Pancreaticography (MRCP) revealed a fistula between the pancreatic duct and the pleural space [Figures 3 and 4]. The patient underwent Endoscopic Retrograde Cholangio Pancreatography (ERCP) with stenting of the pancreatico-pleural fistula. There was an improvement in pleural effusion after the procedure.

## Discussion

Drug-induced pancreatitis (DIP) is less common, with an incidence of 2–5% of reported cases of AP in the general population. In most patients with AP (85–90%), the disease resolves on its own within 3–7 days after stopping the culprit drug.[4,5] The drugs appear to induce pancreatitis by direct or indirect effects. Direct effects include toxic or immune-mediated reactions, while indirect mechanisms consist of ischemia, intravascular thrombosis, and increased viscosity of pancreatic juices. Direct immunological effects of drugs typically manifest within the first month of exposure, while toxic effects (such as with didanosine, pentamidine) become evident after a few months.[6] Imatinib is thought to cause pancreatitis by immune complex-mediated mechanism. The pancreatic toxic effect does not seem to be dose-dependent, occurring mainly within the first weeks of therapy. No medical intervention is required, and withdrawal of the compound can resolve pancreatic enzyme abnormalities within 2 weeks. Elevation of pancreatic enzymes might be an immunoallergic reaction or a marker of the drug bioactivity. Here a close differential diagnosis was AP due to CML itself. Approximately 10% of patients with CML suffer significant GI complications. The more common ones are due to leukemic infiltration of bowel and related structures. Rarely peptic ulcers have been reported as a consequence of hyper histaminemia.[7] The reasons why pancreatitis was unlikely due to CML were either symptom resolution on stopping the definitive drug for CML and the absence of accelerated or blast crisis phase as evidenced by a normal peripheral smear and total count.

Imatinib, a selective inhibitor of the BCR–ABL tyrosine kinase, produces high response rates in patients with CML.[8] The bioavailability of the drug after oral administration is 98%. Imatinib is metabolized in the liver and the major route of elimination being the bile, only a small portion is excreted in urine. The US FDA has approved imatinib for first-line treatment of CML. Severe hepatic adverse event has been reported. If elevations in serum bilirubin greater than three times the upper limit of normal (ULN) or in hepatic transaminases greater than five times the ULN occur, imatinib should be withheld until bilirubin values have returned to less than 1.5 times the ULN and transaminase levels to less than 2.5 times the ULN.[8]

The main toxicity of imatinib is myelosuppression. Pancreatitis as a severe adverse event after imatinib treatment is rare with hardly any reported cases.[9] In a series where toxicity of imatinib given for CML was studied, 26.2% of patients required interruption of imatinib due to severe toxicity. The causes were liver dysfunction (n = 6), nausea (n = 4), multiple organ failure secondary to life-threatening infection (n = 3), persistent neutropenia (n = 2), ileus (n = 2), pulmonary bleeding (n = 1), pancreatitis (n = 1), fluid retention (n = 1), and skin rash (n = 1).[(4)] Gastrointestinal adverse events included nausea (42–71%), diarrhea (30–60%), vomiting (15–56%), abdominal pain (23–37%), increased weight (3–30%), dyspepsia (11–24%), flatulence (16–23%), anorexia (6–17%), constipation (6–15%), and taste disturbance (1–14%). Other toxicities include dermatological toxicity as well as pulmonary mucormycosis.[(10)] Pulmonary alveolar proteinosis has been reported at a dose of 400 mg daily.[(11)] Dosage adjustment during treatment with imatinib includes antifungals, antibiotics, prednisolone and dexamethasone, anticonvulsants, etc.[(12)] The recommended dose of imatinib mesylate is 400 mg/day for adult patients in CML chronic phase and 600 mg/day for CML accelerated phase/blast crisis.
